# Current Nuclear Engineering Strategies in the Green Microalga *Chlamydomonas reinhardtii*

**DOI:** 10.3390/life13071566

**Published:** 2023-07-15

**Authors:** Federico Perozeni, Thomas Baier

**Affiliations:** 1 Department of Biotechnology, University of Verona, 37134 Verona, Italy; 2 Algae Biotechnology and Bioenergy, Faculty of Biology, Center for Biotechnology (CeBiTec), Bielefeld University, 33615 Bielefeld, Germany

**Keywords:** genetic engineering, green microalgae, sustainable bioproduction concepts, recombinant expression, nuclear transformation, *Chlamydomonas reinhardtii*

## Abstract

The green model microalga *Chlamydomonas reinhardtii* recently emerged as a sustainable production chassis for the efficient biosynthesis of recombinant proteins and high-value metabolites. Its capacity for scalable, rapid and light-driven growth in minimal salt solutions, its simplicity for genetic manipulation and its “Generally Recognized As Safe” (GRAS) status are key features for its application in industrial biotechnology. Although nuclear transformation has typically resulted in limited transgene expression levels, recent developments now allow the design of powerful and innovative bioproduction concepts. In this review, we summarize the main obstacles to genetic engineering in *C. reinhardtii* and describe all essential aspects in sequence adaption and vector design to enable sufficient transgene expression from the nuclear genome. Several biotechnological examples of successful engineering serve as blueprints for the future establishment of *C. reinhardtii* as a green cell factory.

## 1. Introduction

Green microalgae offer several key features for application in industrial biotechnology, such as efficient and scalable biomass accumulation, simple and cheap cultivation in minimal salt solutions on non-arable land as well as rapid growth rates [1], fueled by efficient photosynthetic light harvesting [2]. Depending on the respective culture conditions, microalgae harbor great metabolic flexibility and are an exceptional source of valuable compounds, such as proteins, lipids, carbohydrates or pigments. Many green microalgae species are “Generally Recognized As Safe” (GRAS) and selected candidate strains are currently used for bioproduction, such as *Haematococcos lacustris* and *Dunaliella salina* for pigments or *Chlorella vulgaris* and *Scenedesmus obliquus* for lipid biosynthesis. These examples accelerate the development of a sustainable bioeconomy based on microalgae biomass as a renewable and powerful resource.

However, recent developments in genetic engineering and synthetic biology have enabled the design of new and promising production concepts, yielding increased product titers and improved carbon use efficiency. The recombinant biosynthesis of non-native products can be established to create additional layers of value to maximize the utilization of microalgal biomass. Furthermore, it offers possibilities for auxotrophy engineering, which strategically increases the biocontainment of industrial aspects, such as the phosphonate dehydrogenase ptxD [3] or acetate dependency [4]. 

Efficient tools for both nuclear and chloroplast-based transformations have been widely established for several microalgae species [5], with the most developed toolkit available for *Chlamydomonas reinhardtii*. It is an attractive model organism for investigations of photosynthesis, phototaxis and cilia biogenesis and has recently emerged as a promising host for the design of novel bioproduction concepts and the investigation of present microalgal gene regulation. Nuclear transformation of *C. reinhardtii* is technically easy to facilitate, inexpensive and allows targeting of the protein of interest to any cellular compartment. Through efficient genetic domestication [6,7,8], specialized *C. reinhardtii* strains have been designed with an increased capacity for transgene expression. Powerful expression elements including promoters, terminators and their corresponding 5′ and 3′-untranslated regions (UTRs) have been identified for the establishment of strong constitutive or inducible heterologous expression [9]. A breakthrough technology is the synthetic redesign of target gene sequences, which plays essential roles in achieving maximal heterologous gene expression in *C. reinhardtii*. Versatile and standardized vector systems [9] simplify the exchange of genetic parts across the research community and help to assemble even complex expression cassettes. Several selection markers and reporters have been characterized to assist in high-throughput screening for the identification of transformants exhibiting gene expression at relevant levels. Combinations of suitable selection markers allow multiple iterations of transgene integrations (gene stacking) to maximize expression and reconstitute complex metabolic pathways. 

**Figure 1:**
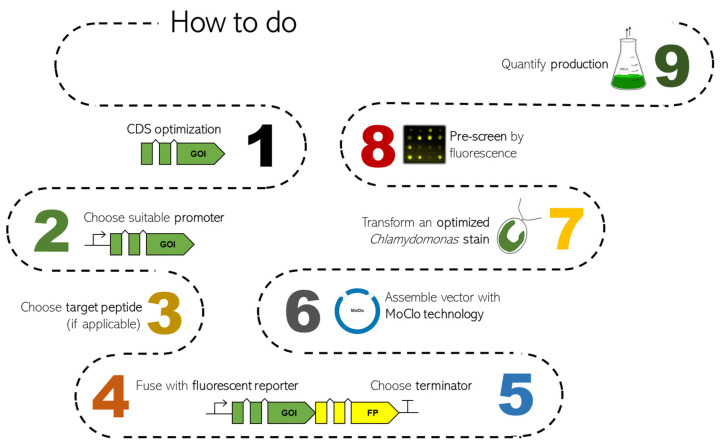
Roadmap overview of the state of the art of *Chlamydomonas* nuclear transformation and optimization of heterologous genes. Each point, with constraints and solutions, will be discussed in the following chapters. In detail: CDS optimization (chapter 1), promoters (chapter 2), target peptides (chapter 3), fluorescent proteins (chapter 4), terminators (chapter 5), MoClo (chapter 6), optimized strains (chapter 7), pre-screening (based on fluorescence measurements) and production quantification (specific to each product depending on individual properties; typically via chromatography or proteomics methods).

These current developments allow the efficient use of eukaryotic microalgae as attractive hosts for biotechnology and pave the way for the design of microalgae as green cell factories. This review aims to summarize recent state-of-the-art strategies for the application of nuclear genetic engineering in *C. reinhardtii* and provides guidance for the design of efficient bioproduction concepts. Figure 1 shows the roadmap of *Chlamydomonas* transformation, indicating the main steps to optimize and elements to choose.

## 2. Strain Domestication for Efficient Nuclear Transformation

*Chlamydomonas reinhardtii* was initially isolated from potato field soil in Massachusetts (US) in 1945. Over the past eight decades, intensive laboratory use has resulted in comprehensive strain domestication and established a variety of mutant cell lines with desirable traits for cultivation, physiological analysis and gene function characterization. This work includes, e.g., the establishment of auxotrophic selection markers, the partial depletion of cell wall layers and random mutagenesis for functional gene knockout. All three endogenous genomes are fully sequenced [10,11] and progress in genetic and metabolic characterization has allowed *C. reinhardtii* to emerge as a powerful model organism and a promising tool for biotechnological applications. The nuclear genome consists of 17 haploid chromosomes with comparatively high GC content (~68%) [10,12], and the respective reference sequence information was recently updated [12]. The stable nuclear transformation of *C. reinhardtii* was reported to be successful using several methods [10,12], including glass bead agitation [13,14], particle bombardment [15], electroporation [16] and agrobacterium-mediated gene transfer [17]. Electroporation typically results in the highest transformation efficiency; however, PEG-mediated glass bead agitation is technically less challenging and more frequently used for cell-wall-reduced strains. The integration of foreign DNA into the nuclear genome occurs at comparably high frequencies and via non-homologous end joining (NHEJ) at random positions. However, large differences in expression strength exist among regenerated individuals from a transformant population, as the integration loci underlie extended eukaryotic gene regulation. Homologous recombination is very rare in *C. reinhardtii* but was recently employed for Clustered Regularly Interspaced Short Palindromic Repeats (CRISPR)-based genome editing. It can be triggered by the presence of double-strand breaks in the respective DNA region [18] and is further supported by cell synchronization [19] and the application of heat shock [20,21]. The unique nuclear genome’s properties only allow limited expression levels, especially for complex transgenes, coding for large fusion proteins or functional enzymes, which calls for essential sequence optimization to achieve efficient transgene silencing machinery, which involves chromatin condensation as a consequence of histone modifications [6,22,23]. Strategically selected UV generated mutations resulted in the design of *C. reinhardtii* strains UVM4/11, which have been successfully identified to allow high-level transgene expression and carry a reduced gene silencing mechanism [6] due to the loss of a sir2-type histone deacetylase (SRTA) [24]. Similar results were observed for the functional knockout of a cytosine-specific DNA methyltransferase (met1) [25], highlighting the impact of engineering epigenetic gene regulation mechanisms for improved gene expression capacities in *C. reinhardtii*. Industrial cultivation at scale calls for robust strains containing an intact cell wall for increased resistance against mechanical shear stress. A cell-wall-containing strain with a high transgene expression capacity has successfully been isolated and characterized as an alternative to established production strains [7,24]. In addition, the biocontainment of engineered transformants can be increased by the establishment of synthetic auxotrophies, such as the ability to metabolize inorganic phosphite via overexpression of the NAD+-dependent phosphonate dehydrogenase ptxD from *Stutzerimonas stutzeri*. These strategies were recently applied in strain UPN22 via the overexpression of ptxD, nitrate reductase (nit1) and nitrate assimilation regulatory protein (nit2), assisting in the cultivation and engineering of *C. reinhardtii* at an industrial level [8].

## 3. Promoters and Terminators

*Chlamydomonas reinhardtii* exhibits unique transcription initiation machinery solely relying on endogenous promoter sequences. Several studies have investigated the application of well-characterized exogenous alternatives, e.g., derived from plant viruses, but failed to establish stable transcription at a high level [17,26,27,28,29,30]. However, the recombinant application of endogenous promoters has successfully been established and is routinely applied for nuclear transgene expression. The ribulose bisphosphate carboxylase small subunit 2 (RBCS2) is the highest expressed gene under vegetative conditions [31] and its upstream region was identified to induce the strong constitutive transcription rates when used as a promoter for transgene expression [32]. Fusions with respective sequence elements from *C. reinhardtii* heat shock protein 70 (HSP70A) promoter further increased transcription and reduced epigenetic gene silencing [33,34]. Currently, the resulting chimeric promoter HSP70/RBCS2 (pAR) is widely applied in nuclear genetic engineering concepts [35,36,37,38,39,40,41,42,43,44]. Recently, rational promoter engineering was performed via strategic sequence modifications of the *C. reinhardtii* βTUB2 promoter, which resulted in the new, synthetic pAßSAP(i) promoter [45], which is currently the strongest promoter available for *C. reinhardtii* and allows product yields four-fold higher compared to other expression elements [45]. 

Sequence characterization from the photosystem I reaction center subunit II (PSAD) gene [46] identified the corresponding upstream region to induce strong constitutive nuclear transgene expression in *C. reinhardtii* and the corresponding N-terminal chloroplast targeting peptide (CTP) to enable post-translational protein import into the chloroplast. In addition, a synergy between the PSAD 5′UTR and its respective CTP has been identified, which depicts a distinct interaction of the applied promoter, 5′UTR and CDS, which influences transgene expression intensity [45]. Moreover, the mRNA folding energy in the translation initiation vicinity significantly affects gene expression [47] for correct ribosome assembly. 

Inducible transcription allows the development of advanced synthetic biology strategies, enables controlled gene expression regulation, and can be essential when gene products cause toxicity. The most applied inducible promoters in *C. reinhardtii* include iron-dependent FEA1 [48], nitrogen-dependent NIT1 [49], alcohol-inducible PalcA [50], salt-inducible GPDH3 [51] and Cu-dependent CYC6 [52]. In addition, the application of a thiamin-dependent riboswitch [53] was successfully established, which further expanded the genetic toolbox of *C. reinhardtii* for tunable expression. Despite these developments, limited gene expression mediated by poor transcription rates remains the major bottleneck in establishing engineered gene expression from the nuclear genome in *C. reinhardtii* and calls for the improved design of strong constitutive alternatives. 

Terminators play a still underestimated role in gene expression regulation as they stabilize mRNA products by inducing polyadenylation and participate in the re-initiation of transcription. Their effect has recently been studied in two systematic investigations [45,54,55]. Two of them underlined the capacity of the endogenous *C. reinhardtii* ferredoxin 1 (FDX1) sequence for the efficient termination of transcription for high transgene expression [45,55], but no strategic sequence optimization has been demonstrated yet.

## 4. Optimization of Transgene Sequences

The nuclear genome of *C. reinhardtii* possesses several unique properties, which call for the customization and adaption of heterologous DNA to function within the endogenous expression machinery. The *C. reinhardtii* coding sequences contain a comparably strong codon bias and high GC content of 68% [10,56]. Codon optimization of transgenes to match the present tRNA pool is a common strategy in genetic engineering in any host and assists in efficient protein translation in *C. reinhardtii* [47,57]. 

Furthermore, endogenous coding sequences are regularly interspaced by introns (~6.4 introns per gene [10]) and the average intron length in pre-mRNA transcripts outcompetes the average exon length (336 bp compared to 224 bp) [10]. Although introns do not contribute to protein translation, they are known to be important elements in gene expression as they enable alternative splicing [58] and have been shown to regulate gene expression [59,60,61] by containing transcriptional enhancers [62] or additional transcription factor binding sites [63], by altering the transcription start site (TSS) [64,65] or by enhancing mRNA stability and export [66,67]. In addition, it was observed that the presence of introns in coding sequences induces an effect called “intron-mediated enhancement” (IME), which stimulates the expression of the originating transgene in feedback regulation and further complicates eukaryotic gene expression regulation. The effect of the synthetic integration of several endogenous and exogenous introns has been systematically characterized in *C. reinhardtii* [41,67,68,69] and the first intron from RBCS2 (RBCS2 intron 1) is commonly used for the synthetic adaption of heterologous DNA [36,37,42,43,44,70]. It is likely that a reduced exon length, spliceosome processing and sequence specific regulation assist in the successful expression of fully optimized transgenes, and this is an essential step for successful transcription continuation in *C. reinhardtii*. 

The recently developed online web tool Intronserter (https://bibiserv.cebitec.uni-bielefeld.de/intronserter (accessed on 14 July 2023)) allows the convenient redesign of any target sequence [71] and will help to establish *C. reinhardtii* as a green cell factory.

## 5. Selection Markers and Reporters for Gene Expression

Nuclear transformation typically employs the co-integration of a selectable marker along with the desired gene of interest. It confers the ability to grow in the presence of selective agents to successfully transform cells for isolation from the initially applied biomass. Two major strategies for positive selection are well established for *C. reinhardtii*, either to restore vegetative growth by complementing an existing auxotrophy, or via the expression of proteins that inactivate selective antibiotics or herbicides (Table 1). The most common auxotrophic markers involve mutations in the endogenous argininosuccinate lyase (*ARG7*) [72], N-acetyl ornithine aminotransferase (*ARG9*) [73] or nitrate reductase (*NIT1*) [14], prohibiting the biosynthesis of arginine or nitrite, respectively. Recently, the *C. reinhardtii* spermidine synthase (*SPD1*) was confirmed to be essential for the polyamine biosynthesis pathway in *C. reinhardtii* and was successfully established as a powerful new auxotrophic marker with versatile biotechnological applicability [21]. Auxotrophic mutant cell lines require the appropriate supplementation of essential metabolites via a culture medium for survival, and prototrophy can be restored by complementation with the functional CDS of the intact gene. However, differences in expression strength in selected transformants can induce variable supply of the respective metabolites, which may result in inefficient growth and complicates comparisons of complemented and supplemented cultures.

The majority of genetic engineering attempts rely on the use of selection markers that confer resistance against functional antibiotics or herbicides via detoxification. The most commonly used genes are the aminoglycoside 3′-phosphotransferases *aphVIII* from *Streptomyces rimosus* [74,75] and *aphVII* from *Streptomyces hygroscopicus* [76], the bleomycin-resistance protein (*shble*) from *Streptoalloteichus hindustanus* [77,78] or aminoglycoside (3″) (9) adenylyltransferase (*aadA*) from *Escherichia coli* [79,80]. However, several other selection systems have successfully been established (e.g., the NADP-requiring oxidoreductase *TetX* or nourseothricin N-acetyltransferase (*NAT*)), and several combinations can be applied for iterative transformations (Table 1). 

**Table 1:** Selectable markers. List of several commonly used selection markers for selection after nuclear transformation of *C. reinhardtii*. Table is organized by type of selection marker: Auto (autotrophy), AB (antibiotics) and Herb (herbicide).

Type	Gene	Screening Mechanism	References
Auto	*ARG7*	Growth in arginine-free medium	[72]
Auto	*NIT1*	Growth in ammonium-free medium	[14]
Auto	*SPD1*	Growth in spermidine-free medium	[21]
AB	*aphVII*	Resistance to hygromycin B	[76]
AB	*aphVIII*	Resistance to paromomycin, neomycin and kanamycin	[74,75]
AB	*Shble*	Resistance to bleomycin and sapromycin	[77,78]
AB	*aadA*	Resistance to spectinomycin and streptomycin	[79,80]
AB	*NptII*	Resistance to paromomycin, neomycin and kanamycin	[81]
AB	*TetX*	Resistance to tetracycline	[82]
AB	*NAT*	Resistance to nourseothricin	[83]
AB	*CRY-1*	Resistance to cryptopleurine and emetine	[84]
AB	*BSR*	Resistance to blasticidin S	[85]
Herb	*GAT*	Resistance to glyphosate	[86]
Herb	*PDS (R268T)*	Resistance to norflurazon	[86]
Herb	*protox rs-3*	Resistance to oxyfluorfen	[86]

**Figure 2:**
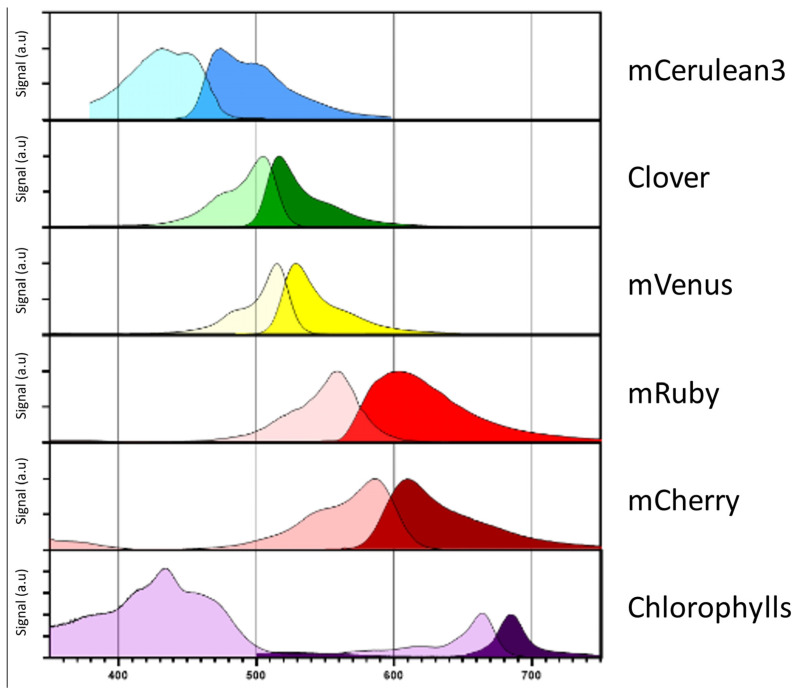
Fluorophore absorption and emission spectra. Absorption (light color) and emission (dark color) spectra of the most commonly employed fluorophores mCerulean3, Clover, mVenus, mRuby and mCherry. Respective spectra were compared in terms of absorbance and emission to native chlorophylls in *C. reinhardtii*. The respective coding sequences for these fluorophores are included in the current MoClo toolkit [9] as well as part of the pOptimized vector system [92]. The respective information was derived from FPbase (www.FPbase.org).

During nuclear transformation, transgenes are randomly integrated via non-homologous end joining into introduced chromosomal double-strand breaks. Differences in eukaryotic gene regulation at the respective integration site greatly influence the expression strength via “position effects” and lead to high variability in target protein accumulation within the individuals of a transformant population. For expression quantification, target proteins are typically fused to suitable fluorescent reporters. Several engineered variants of the *Aequorea victoria* green fluorescent protein (GFP) [87] exist, which possess absorption and emission characteristics that do not overlap with native chlorophyll or carotenoid signals in *C. reinhardtii* [88,89,90]. Additionally, several alternative, red fluorescent proteins derived from coral anemones were designed to complement the fluorescent reporter toolkit, e.g., mCherry [91] or mRuby [92,93]. The rapid and non-invasive screening of a multitude of putative transformants directly on the initial transformation plate allows the identification of cell lines with the highest expression [94] and sufficient product yields for biotechnological use. The absorption and emission spectra of the most commonly used fluorescent reporters are shown in Figure 2. Recently, a systematic study investigated further alternatives to these established fluorescence proteins (FP) and proposed specific combinations that enable the detection of up to five independent FP signals from cyan to far-red in living microalgae at the agar plate level and also in protein electrophoresis gels [95].

For secreted proteins, fusions with luciferases have been well established, which emit bioluminescence upon the oxidation of a corresponding substrate (e.g., luciferin or coelenterazine). They offer higher sensitivity and signal intensity compared to other reporter systems; however, their detection typically cannot be applied in vivo. The most popular luciferase is the gLuc from the marine organism *Gaussia principeps* [92,96]. Recently, a new luciferase-based system called NanoLuc was developed by engineering both enzymes and substrates to improve the luminescence intensity ~150-fold compared to established luciferases [97].

## 6. Vector System

The ease of nuclear transformations allows high screening throughput for efficient identification of successful target protein accumulation. Over the past few decades, numerous genetic building blocks coding for functional expression elements have been characterized (e.g., promoters, reporters, selection markers and coding sequences) and made available in public databases for the strategic customization of expression constructs (e.g., *Chlamydomonas* Resource Center, Addgene). Innovative vector systems are required to facilitate the desired construct modifications and the rapid assembly of complex constructs. The first comprehensive attempt to standardize the available *C. reinhardtii* toolkit was the development of the pOptimized vector system [92]. Each expression element position can be exchanged via unique type IIR restriction enzyme sites, which allows the direct exchange of each genetic part using classical cloning methods. The pOptimized system was effectively applied for terpene bioproduction and several versatile modifications exist [36,37,43,44,70].

**Figure 3:**
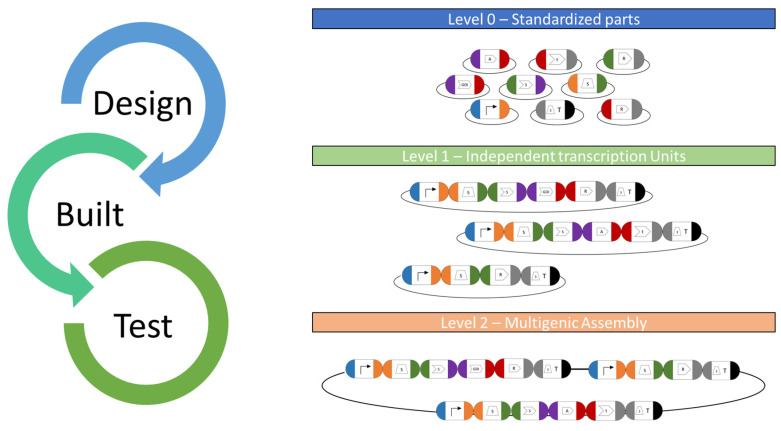
MoClo toolkit workflow. Basic and standardized parts cloned into level 0 plasmids serve as libraries for all available genetic parts for *C. reinhardtii* nuclear engineering. They serve as available resources for potential combination in functional transcriptional units (TU) for genetic engineering. For this, the respective plasmids will be digested and suitable DNA parts ligated using type IIS restriction enzymes and ligases in a “one-pot” reaction. The respective fusion sites are specific to each position in a designed ORF (indicated by colors) and allow correct orientation during TU assembly in an acceptor vector. Several TUs can be combined into a level 2 multigene expression vector (device), which allows the de novo design of complex expression vectors from scratch and the co-expression of several GOIs along with a selection marker.

More recently, an alternative system based on the MoClo syntax was designed, which employs standardized sequence overhangs and the Golden Gate assembly technology [9]. MoClo toolkits are well established in *Escherichia coli* [98,99], *Saccharomyces cerevisiae* [100], mammalian systems [101], cyanobacteria [102] and plants [103], and they allow the rapid de novo assembly of any designed construct in a “one-pot” reaction. A recently developed *Chlamydomonas* MoClo toolkit (CrMoClo) provides 119 genetic parts for basic construct designs [9] and it is being further expanded by several projects [42,45,85,104,105]. The available level 0 parts can be strategically combined using type IIS restriction enzymes for the fully customized design of level 1 transcription units (module) and further into a level 2 multigene expression vector (device). It allows the de novo design of complex expression from scratch and greatly assists in synthetic biology approaches using *C. reinhardtii* (Figure 3).

## 7. Biotechnological Application

Several studies have successfully demonstrated the use of optimized transgenes for the efficient nuclear engineering of *C. reinhardtii* and achieved the industrially relevant bioproduction of valuable products, including terpenoids, polyamines, recombinant proteins and pigments. Terpenoids are structurally complex molecules with a broad range of biotechnological applications, e.g., as biopharmaceuticals, cosmetics and natural flavoring molecules [106,107,108,109,110]. The first examples of engineered light-driven terpenoid production from *C. reinhardtii* achieved 0.5 mg/L (0.92 ± 0.24 µg/g CDW) of the scent molecule patchoulol [43], 11 mg/L (10.3 ± 0.7 mg/g CDW) of the biodiesel precursor (E)-α-bisabolene and 50 mg/L (80 mg/g CDW) of the biopharmaceutical precursor 13R(+) manoyl oxide [44]. Engineering achievements indicate the powerful carbon flux and fundamental plasticity of the plastid-located 2-C-methyl-d-erythritol 4-phosphate/1-deoxy-d-xylulose 5-phosphate (MEP) pathway in *C. reinhardtii* [111]. It is an exceptional source of sustainable metabolites [36,111] and provides abundant precursor isopentenyl pyrophosphate (IPP) and dimethylallyl pyrophosphate (DMAPP) for engineered isoprenoid biosynthesis [43,44,45,106,107,108,112,113]. Recently, sophisticated metabolic engineering has been conducted to eliminate present bottlenecks from the MEP pathway via the overexpression of strategically engineered fusion proteins for increased flux towards terpenoid products [42]. Phototrophic cultivation in high-cell-density media [21] yielded 656 mg/L (200 mg/g CDW) of the fine chemical and perfume ingredient sclareol [42]. In addition, engineered perturbations of the downstream carotenoid pathway through ketocarotenoid biosynthesis increased the flux from the MEP pathway and enhanced the production of the natural rubber component isoprene to 362 mg/L [114].

The modern chemical industry calls for new, resource-efficient and sustainable value chains for the production of key base chemicals as valuable resources [115]. Polyamines, such as putrescine and cadaverine, serve as versatile building blocks for the synthesis of polyamides, linear polymers with excellent durability and strength properties for textiles (e.g., nylon) and industrial as well as household utensils. Bio-based production of these base chemicals has effectively been shown using engineered *C. reinhardtii* [115,116]. This work included the systematic screening and overexpression of functional amino acid decarboxylases in combination with the application of high-cell-density cultivation. Under phototrophic conditions, production of up to 0.24 g cadaverine/L was achieved, with maximal productivity of 0.1 g/L/d [115]. Similar amounts (0.2 g/L) were quantified of the diamine putrescine after the overexpression of an ornithine decarboxylase from *Atropa belladonna* and the genome-editing-based inactivation of putrescine degradation via amine oxidation (amine oxidase 2, AMX2) in *C. reinhardtii* [116]. Interestingly, optimized transgenes expressed to a high level from a single transformation event and iterative transformations had only a minor effect on product accumulation, which indicates high metabolic turnover rates in microalgae. Both examples demonstrate the potential of the CO_2_-based bioproduction of polyamine base chemicals and promote the sustainable utilization of *C. reinhardtii* engineering concepts for modern bio-industry.

The secretion of valuable proteins into the culture supernatant is an attractive strategy to create another layer of value besides the utilization of microalgal biomass. Engineered fusion proteins were successfully designed to enable the efficient secretion and purification of human epidermal growth factor (hEGF) from *C. reinhardtii* culture media [40]. Secreted hEGF reached concentrations of 100 µg/L after 48 h and exhibited full biological activity compared to commercial standards. Recently, the SARS-CoV-2 spike protein was shown to accumulate up to 11.2 µg/L in *C. reinhardtii* culture supernatants [117]. Engineering of the C-terminus of secreted proteins effectively assisted in transport through the secretory pathway [40,118] and resulted in increased reporter protein accumulation of up to 15 mg/L [118]. Recombinant protein secretion suffers from target protein complexity (e.g., glycosylation and disulfide bond formation) and further research is necessary to increase the yields from nuclear engineering attempts.

*C. reinhardtii* has developed a powerful carotenoid pathway to cope with fluctuating and high light intensities. It harbors an evolutionary silenced ß-carotene ketolase (BKT), which was successfully expressed upon transgene optimization and reintegration into the nuclear genome [37]. It induced a noticeable color change from green to red and allowed the accumulation of up to 4.3 mg/L/day of ketocarotenoids, which are typically absent in *C. reinhardtii*. These pigment alterations did not affect growth under vegetative conditions [37]. In contrast, engineering of astaxanthin accumulation reduced photoinhibition and increased biomass productivity under very high light intensities [119], likely due to the reduced cellular chlorophyll content, increased ROS scavenging capacity and antioxidant activity. The biotechnological production of astaxanthin was recently complemented by a systematic metabolic engineering approach to overcome rate-limiting steps in the carotenoid biosynthesis pathway in *C. reinhardtii*. The combined overexpression of BKT, ß-carotene hydroxylase and phytoene synthase enabled the accumulation of 23.5 mg/L, with maximal productivity of 1.09 mg astaxanthin/L/h [36], which was assisted by the application of high light intensity under phototrophic conditions. Astaxanthin production in engineered *C. reinhardtii* is favorable due to its increased extractability and bioaccessibility as a result of the lack of rigid cell walls, and it might compete with native production in *Haematococcus lacustris* when the yields are sufficient [37].

These examples demonstrate the present capacity of nuclear engineering and the great biotechnological potential of *C. reinhardtii* as a powerful green cell factory. 

## 8. Conclusions

Recent progress within the scientific community has improved the capacity to express transgenes from the nuclear genome of *C. reinhardtii*, depicting this green microalga as a promising chassis for biotechnology. Combined efforts for transgene optimization and state-of-the-art nuclear engineering strategies have been summarized and the given examples demonstrate the efficient use of *C. reinhardtii* for the synthesis of valuable bio-products at levels comparable to those of established hosts and serve as blueprints for future applications. However, further research is necessary to fully elucidate the present microalgal gene expression regulation, to further increase production titers in engineered cell lines and to allow industrially relevant cultivation at scale for efficient use as a green cell factory.
